# Vegetable Fibers in Cement Composites: A Bibliometric Analysis, Current Status, and Future Outlooks

**DOI:** 10.3390/ma18020333

**Published:** 2025-01-13

**Authors:** A. Arvizu-Montes, M. J. Martinez-Echevarria

**Affiliations:** Department of Construction Engineering and Projects of Engineering, University of Granada, 18071 Granada, Spain; mjmartinez@ugr.es

**Keywords:** vegetable fibers, cement composites, durability, mechanical properties, scientometric analysis

## Abstract

The use of vegetable fibers (VFs) in cement-based composites has increased in recent years owing to their minimal environmental impact and notable particular properties. VFs have aroused interest within the scientific community because of their potential as a sustainable alternative for construction. This study presents a comprehensive bibliometric analysis of VFs in cement composites using data from the Scopus database and scientometric tools to explore publication trends, influential sources, and research directions. Key findings reveal a steady increase in publications, with *Construction and Building Materials* identified as a leading journal in the field and China and Brazil as prominent contributors in terms of publications and citations. The analysis highlights a strong focus on mechanical properties and durability, reflecting the interest of the scientific community in optimizing VF composites for construction. Furthermore, this study includes a revision of the most influential studies addressing VF classification, durability improvements, and advanced applications of VFs in building applications. Finally, future research opportunities are outlined, emphasizing Life Cycle Assessment (LCA), industry integration, CO_2_ absorption, and the application of machine learning techniques to advance the development of VF composites. This work provides a comprehensive overview of the field, suggesting future guidelines and promoting collaborative research.

## 1. Introduction

In the past few decades, there has been an increasing interest in researching and developing sustainable materials within the building sector [1,2,3]. This attention is driven, among other factors, by the current environmental situation, given that construction is acknowledged as one of the most ecologically impactful industries globally [4,5]. According to McLellan et al. [6], cement production alone generates between 5 and 7% of total CO_2_ emissions worldwide, standing as one of the main contributors to greenhouse gas release. Furthermore, it is well known that cement-based materials are susceptible to deterioration, which may result from multiple factors, including inadequate design, mechanical, physical and chemical effects, or the passage of time itself, compromising their service life [7]. Several alternatives, such as the inclusion of additives or admixtures in the matrix, have been studied to mitigate these obstacles [8].

For instance, Chen et al. [9] highlighted the fact that integrating urban waste in concrete provides not only technical benefits but also environmental advantages. This approach minimizes greenhouse gas emissions and decreases the ecological impact of concrete production by replacing raw materials and admixtures essential for concrete manufacturing with waste, thereby resulting in economic advantages.

In this context, a technique that has gained recognition involves the incorporation of fibers as reinforcement [7,10], as they can improve the mechanical behavior of composites and decrease the cement content without compromising their properties, thus resulting in materials with less environmental impact [11]. Commonly, the most prevalent fibers employed in construction originate from steel and synthetic materials [12]. However, these fibers are not economically affordable, and their production generates CO_2_ emissions as well as the consumption of non-renewable resources [13]. Hence, extensive research has been dedicated to studying other types of fibers, highlighting those derived from natural sources.

Natural fibers have been employed by humans since ancient times for diverse applications, including their inclusion in composites [14]. These types of fibers can be broadly classified according to their origin: (i) animal fibers (e.g., wool), (ii) mineral fibers (e.g., basalt), and (iii) vegetable fibers (e.g., sisal) [15]. In particular, this study discusses the potential of vegetable fibers (VFs).

In light of both the growing ecological need for “eco-friendly materials” and the drawbacks previously discussed, natural fibers derived from plants emerge as a solid contender for reinforcement in cementitious materials. This is mainly attributed to their cost-effectiveness, minimal environmental impact, and global availability [16], as well as their light weight and notable tensile strength [17,18], contrasting favorably with conventional reinforcements.

Composite materials are continuously advancing in various fields, and in this context, the development of construction and cement matrices is not lagging behind [19]. Likewise, the scope of studies on VFs is broad, covering research on hemp, flax, sisal, abaca, alfa, jute, and diverse other vegetable fibers. Generally speaking, the addition of fibers aims to prevent and regulate the tensile cracking of these materials; however, they can also be incorporated to improve other properties, like thermal insulation [20] or impact strength [21]. Nevertheless, additional studies suggest that the structural composition of VFs, characterized by both crystalline and amorphous regions [22], exhibits limitations for composites, including porosity and moisture absorption [22,23], leading to poor fiber–matrix bonding. These factors, among others, are acknowledged to significantly weaken the overall performance of composite materials, thereby resulting in reduced durability and mechanical behavior.

To comprehensively address the multifaceted dimensions of including VFs in cement-based materials, scholars have undertaken extensive reviews of the existing literature, discussing the topics inherent in this scientific field. These works identify solutions to specific questions and challenges within this domain. Nonetheless, it is crucial to acknowledge that this approach may be influenced by subjective factors based on the selected information or data incorporated into the research, as well as potential exclusions [24]. In this context, despite finding numerous reviews and experimental papers focused on VFs, it is particularly noteworthy that the use of a science mapping method is not widespread in databases, in comparison to reviews. The science mapping approach offers a powerful tool to visualize and browse complex research fields, providing insights into evolving concepts that traditional reviews do not always take into account [25].

**Figure 1:**
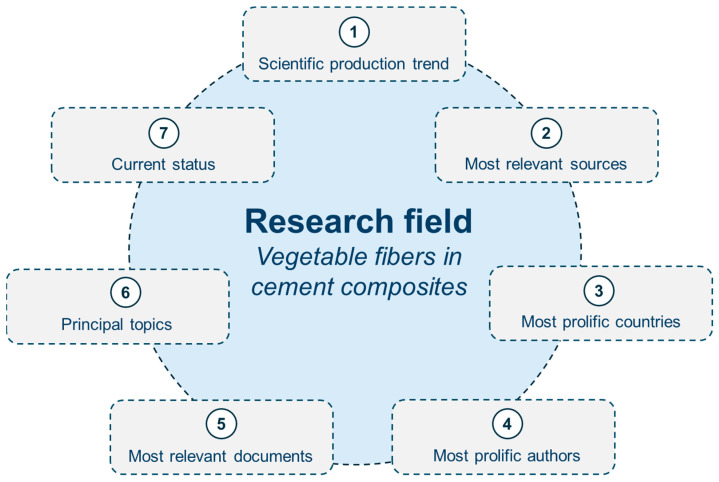
Inquiries to be addressed regarding the research field.

In essence, reviews systematically synthesize and organize research to reveal connections and opportunities, while science mapping effectively visualizes the relationships and trends within a given field of study. Additionally, Khan et al. [26] state that a scientometric analysis can quantitatively evaluate large numbers of bibliometric data supported by specialized software, whereas conventional reviews may have a limited capacity to link diverse areas of the literature comprehensively. Therefore, the initial phase of this investigation aims to evaluate the research field related to vegetable fibers, supported by a bibliometric analysis. The inquiries that arise from this assessment and which it aims to address are visually represented in Figure 1.

After the assessment, this study aims to construct a comprehensive overview of the current status regarding the application of VFs in cement composites for building purposes. Subsequently, this analysis will explore future directions, providing novel insights for advancing research and development in fiber-reinforced materials. The identified future outlooks aim to innovate the application and understanding of vegetable fibers in construction materials.

The overall structure of this paper comprises six sections, with the Introduction being the first. The second section details the methodology employed in this study. The third chapter presents a scientometric analysis outlining the most significant results of the assessment and their corresponding discussion. Following this, a concise review of the most relevant documents briefly describes the current status of research. The next chapter explores future outlooks on upcoming trends and research directions. Finally, the conclusions drawn from the research are presented.

## 2. Methodology

A bibliometric analysis could be conceived, at its core, as the application of quantitative techniques to bibliometric data [27]. This methodology allows for the exploration of a wide range of publications from different sources and countries [28], which turns it into an effective tool for academics to take preliminary steps in establishing an overview of a determined research field. So, the current study uses a science mapping approach, analyzing the available literature regarding the use of vegetable fibers in cementitious matrices.

Prior to commencing the assessment, it was necessary to select the appropriate database for this research. Scopus and Web of Science are two of the most well-known databases for searching publications, including different scientific fields; however, according to previous authors, Scopus is a strongly suggested search engine [29], as it covers more journals and has a larger number of documents that are not included by Web of Science [30,31]. Additionally, as stated by Afgan and Bing [32], Scopus provides an updated bibliometric data list and greater compatibility with VOSviewer compared to Web of Science. Therefore, for the present analysis, the authors decided to retrieve the data from Scopus.

Data collection from Scopus was conducted in February 2024. This database includes publications from all over the world; hence, it is possible to find content published in different languages [33]. Given that English is the predominant language of scientific publications and, aiming to standardize the information further analyzed, only documents published in English were selected for this study. Also, the term “natural fibers” is commonly used by authors who work with VFs [34]. However, as explained in the previous chapter, and based on observations made during multiple searches, this term might include documents pertaining to different types of fibers outside the scope of this assessment. Therefore, after some inquiries, the definitive search string applied using Boolean operators was “vegetable OR plant AND fiber OR fibre AND concrete OR mortar”, considering a search within “Article title, Abstract, Keywords”, giving a result of 1205 documents. The first step in discarding unnecessary records was to restrict the search in accordance with the limits provided by Scopus. The filters selected during the data retrieval are listed in Table 1.

In this type of research, it is crucial to consciously clean the acquired information to avoid duplication or inaccurate data [27]. Thus, after the filters were applied, a detailed revision was performed, and it was necessary to manually remove some documents that did not align with the scope of this study. This procedure was conducted by analyzing both the titles and the abstracts of the extracted data to evaluate whether those papers constituted research works that matched the objectives of this bibliometric analysis. For instance, some papers retrieved from the dataset focused on fibers beyond the scope of this analysis, such as basalt or glass fibers. Additionally, the inclusion of the term “plant” in the search string resulted in unrelated documents, such as those discussing nuclear plants or industrial energy facilities. Consequently, after this manual removal, the second cleaning provided 240 publications for evaluation.

**Figure 2:**
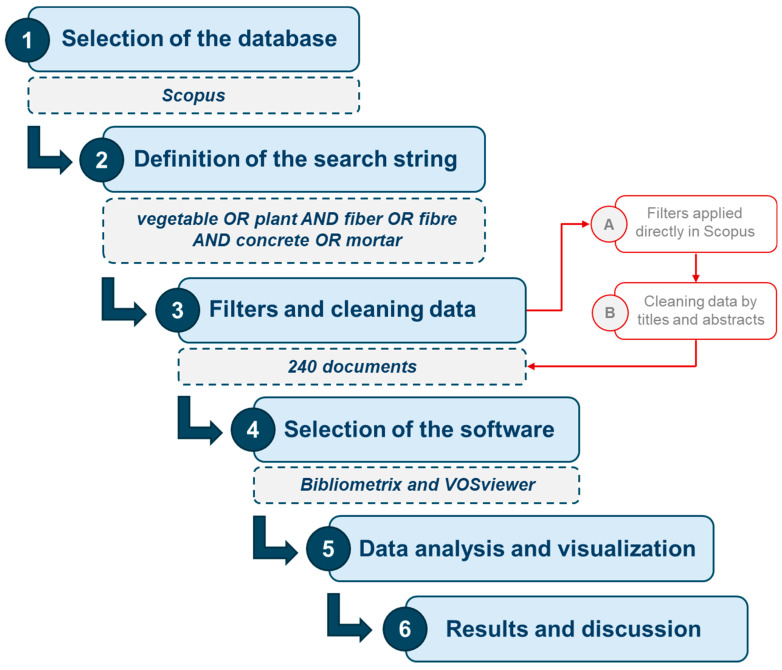
Workflow followed to conduct the bibliometric analysis.

The results obtained were exported in two different types of files: BibTex and CSV (comma-separated values). Moreover, supporting thesaurus files were created to eliminate duplications and standardize concepts, significantly improving the efficiency of the analysis process, particularly with author contributions and keywords. The software chosen to run the analysis were the open-source tools Bibliometrix R-package and VOSviewer (version 1.6.20). Bibliometrix was developed by Massimo Aria and Corrado Cuccurullo, offering a collection of tools to perform bibliometric analysis and data visualization [35], and complemented by biblioshiny, also provided by Aria and Cuccurullo in R-language, using which the analysis could be executed on an interactive web interface [24]. Similarly, VOSviewer is a program introduced by Nees Jan van Eck and Ludo Waltman, with special attention to the graphical representation of bibliometric maps, allowing for their full detailed examination [36]. Finally, in order to visually represent the workflow we followed, Figure 2 summarizes the methodology conducted in the present research.

## 3. Scientometric Analysis: Results and Discussion

### 3.1. Annual Distribution of Publications

**Figure 3:**
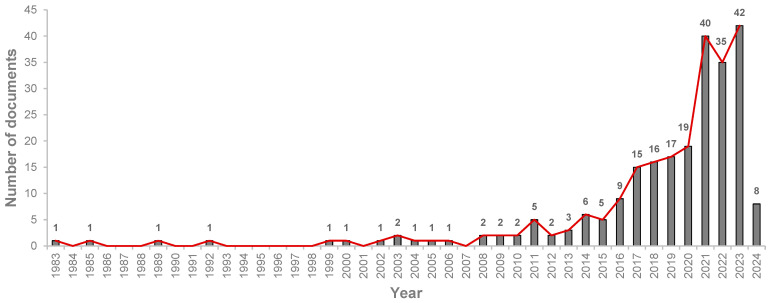
Documents published per year from 1983 to February 2024.

The number of publications registered per year in a determined field of study can be considered the course of growth concerning that scientific topic [37]. Figure 3 illustrates the progress of papers published by year until the day of data retrieval. Initially, the assessment period began in 1993; however, owing to a lack of documents from 1993 to 1998, the range of time was re-evaluated. Extending the timeframe back to 1983 allowed us to incorporate other publications, thereby enriching the scope of this analysis. This 40-year period included earlier influential works and gaps that helps to reflect changes in technology and methods, which simultaneously reveal wider long-term trends and key milestones for the scientific field and its development.

As can be seen from Figure 3, the yearly publication trend demonstrates a progressive increase in the number of publications during the last 10 years or so. Given that reducing the environmental impact of construction through innovative approaches is crucial for sustainable development [38,39], this increase can be attributed to different aspects, such as the growing global focus on sustainability, or the industry’s increased adoption of scientific research. A similar trend was found by Cândido et al. [40] after analyzing sustainable transitions in the construction sector, confirming the importance of sustainability research in construction, which the European Union identifies as a key sector for addressing climate change challenges. Similarly, Ferreira et al. [41] highlighted the growing focus on sustainable construction materials, emphasizing renewable and recycled fibers, noting that the increasing interest in fibers in civil construction aligns with the growth in research over the years.

Nevertheless, a particular exception was noted in 2022, when the growth presented a drop. While fluctuations in research output are common, several factors may have contributed to this decline. For instance, the global disruptions caused by COVID-19 have affected various aspects of academic activity. Delays in research projects, reduced funding, and interruptions in publication processes may have played a role, as fields not directly related to the pandemic were deprioritized [42].

In the same line, this global health crisis led to the cancellation or postponement of scientific conferences. Nicola et al. [43] highlight that these events are crucial for scientific research in many disciplines and promote collaboration and job opportunities among academics. Although some were moved to virtual conferences, they turned out to be less effective in networking and communication. Furthermore, as noted by Rodrigues et al. [44], researchers faced significant changes to their income and had minimal access to traditional workplaces during this period, as many institutions suspended on-site operations.

A study conducted by Gao et al. [45] revealed that, although scientists rejoined their research activities after the lockdown restrictions, they were less likely to pursue new research projects, possibly indicating longer-lasting effects of the pandemic. The temporary stabilization observed in 2022 might reflect the ongoing adaptation of scientific community to the new normal and global recovery effects. This could explain why, in 2023, the number of publications exceeded the previous peak (40 papers), reaching 42 documents. Based on these results and the fact that eight publications were already recorded by February 2024, it is expected that the rise in this scientific topic will continue to grow.

### 3.2. Most Relevant Sources

**Figure 4:**
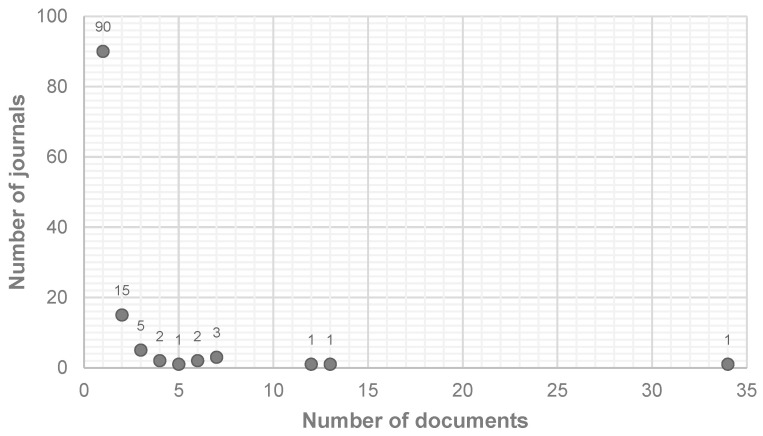
Relationship between published documents and number of journals.

**Figure 5:**
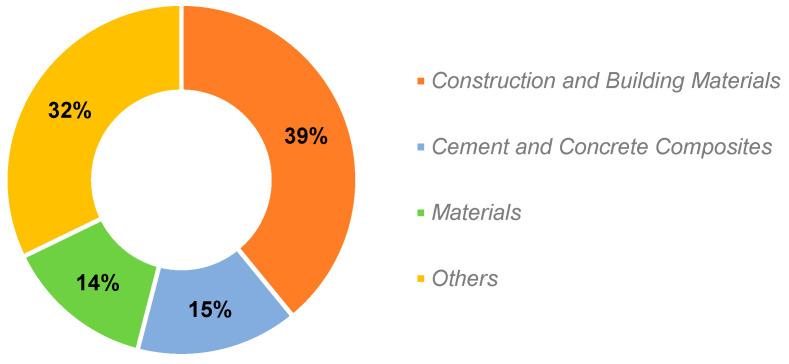
Distribution of documents in the most prolific journals.

**Figure 6:**
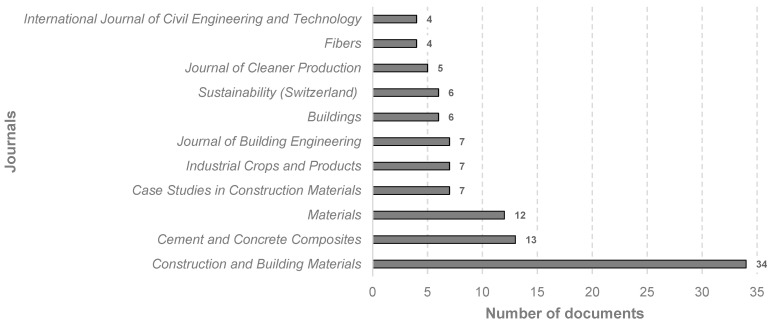
Journals with 4 or more documents in the current study, from 1983 to February of 2024.

The analysis gathered a total of 121 sources. The relationship between the number of publications and the number of journals is represented in Figure 4. As can be noticed, there are ninety journals with only one document each published in the field; on the other hand, one journal has thirty-four publications. Figure 5 illustrates the sources with more than 10 papers, showcasing the distribution of the documents considered for this study, with a clearly superior occurrence of *Construction and Building Materials*, at 39%. Therefore, and considering the high dispersion in the results discussed earlier, it was necessary to contemplate journals with four or more publications in order to achieve a more representative breakdown of the most relevant sources in this topic. This resulted in eleven journals, which are outlined in Figure 6.

The dominance of *Construction and Building Materials* can be attributed to its comprehensive scope, which includes a wide range of construction materials and technology, particularly in areas closely aligned with VF research, such as sustainable and recycled materials. Its strong focus on innovative research in these areas, combined with its high impact and broad academic readership, emphasizes its prominence in the field.

An alternative comparison proposed in this assessment was to cover two different journal-based metrics (JBMs) for the previously considered sources. First, since the information used for this paper was retrieved from Scopus, CiteScore, one of the three parameters found directly from this database, was the first metric included. On the other hand, the second metric incorporated was the Journal Impact Factor (JIF), obtained from *Journal Citation Reports*.

Previous authors have studied these systems of measuring sources. According to Okagbue and Teixeira Da Silva [46], CiteScore could be a more functional metric when compared to the Journal Impact Factor. However, given that these journal-based metrics are founded on similar principles, and considering that the JIF has been in use for a longer period of time and is a recognized indicator within the academic field [47], it was decided to include both metrics, as can be noticed in Table 2. Finally, a relevant finding was that, despite the fact that the *International Journal of Civil Engineering and Technology* is part of the top eleven sources considered in Figure 6, it is no longer included in Table 2, as it was discontinued from Scopus in 2019.

### 3.3. Countries Scientific Production

Analyzing scientific production by country is another approach that can reflect the progress of this research field, bearing in mind the inherent variation in vegetable fibers across the globe. Thus, a total of 59 countries were included in the analysis. In this case, the number of occurrences of each country, considered frequency, was determined after counting the appearances of authors according to country affiliation, which means that each publication is attributed to the countries of all its co-authors.

**Figure 7:**
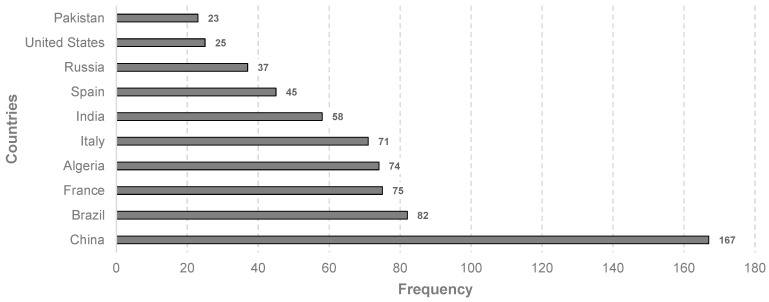
Top countries contributing to the research field, showing regional productivity.

Figure 7 illustrates the top ten countries based on frequency, with a distribution of four European countries (France, Italy, Spain, and Russia), three from the Asian continent (China, India, and Pakistan), two American countries (Brazil and the United States), and only one from the African continent (Algeria). The evidence clearly shows that China is the leading producer of scientific output, with a 51% advantage over its closest counterpart, Brazil, demonstrating its solid leadership in the field over other nations when this metric is considered.

**Figure 8:**
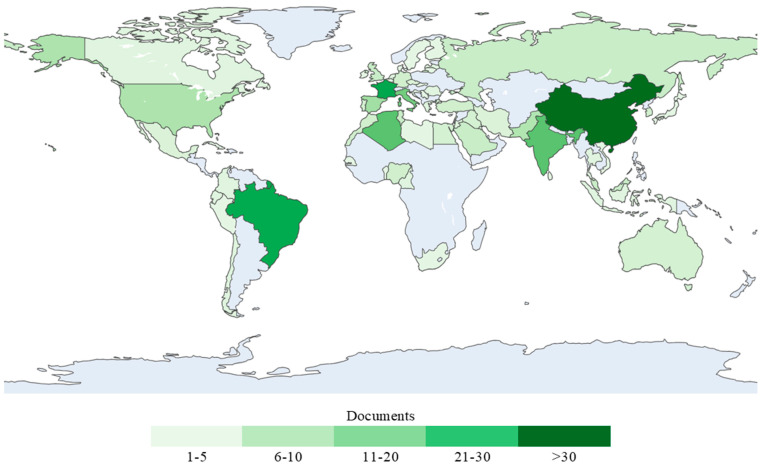
Global distribution of countries by number of published documents.

**Figure 9:**
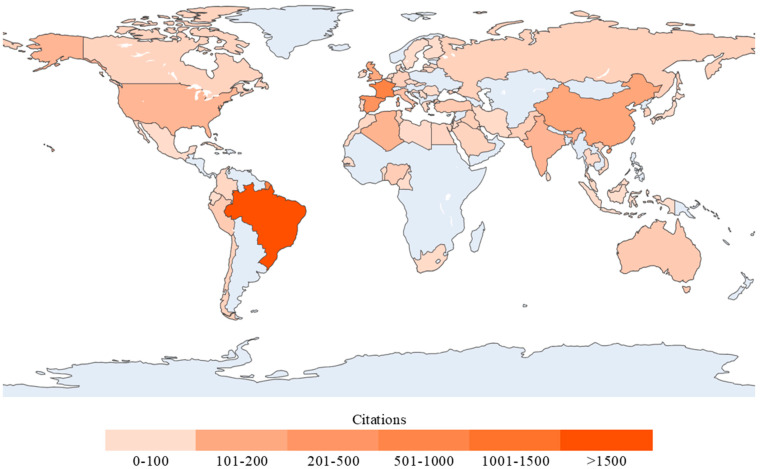
Global distribution of countries by citation impact, reflecting research influence.

Additionally, in a parallel procedure to ascertain which countries are the most productive in this scientific topic, both the quantity of documents and citations from each country were measured. To visually represent these parameters, Figure 8 and Figure 9 distribute the results of the dataset into a worldwide choropleth map, with the number of publications illustrated in Figure 8 and the number of citations in Figure 9. It is well known that scientific production in China has been growing quickly across different disciplines [48,49,50]. Furthermore, prioritizing investments in waste disposal initiatives, China has undertaken substantial measures in solid waste management, as highlighted by Li et al. [51]. These efforts are in line with the research production trend observed by Ferreira et al. [41], which may explain the leadership of this country in terms of documents in this research field.

However, the case of Brazil presents a different dynamic. While this country shows fewer publications than China, it has a higher citation impact, which may reflect the quality, relevance, and influence of the studies published. This trend can be associated with the abundant availability of these types of fibers in the country, enabling their widespread utilization as resources [34,52]. Moreover, García et al. [53] recognized Brazil as the main contributor of studies related to this field from the Central and South American region, with research encompassing 10 of the 19 different types of VFs identified.

In contrast, regions like Africa show a limited scientific output in this field, despite the availability of VFs. Confraria et al. [54] stated that scientific institutions in many African nations face a shortage of skilled researchers, fragmented funding across uncoordinated ministries, and a reliance on intermittent external support with short-term objectives. This situation is reflected in broader trends, as Sooryamoorthy [55] noted that sub-Saharan countries represent less than 1% of global research production. These findings underscore the need for targeted investments and policies to strengthen the research capacity in under-represented regions.

Similarly, to identify the most influential countries, Table 3 ranks the top ten nations, first in terms of the number of publications and second according to the number of citations. To establish a correlation, the average number of citations was included, which corresponds to the calculation of the ratio between citations and documents. This table is quite revealing in several ways. On the one hand, China leads the ranking in terms of the number of documents published, with 38 articles; however, the outcome regarding citations is different, as the highest position is taken by Brazil, with a value of 2339, displacing China to the fourth position, below Spain and France.

An interesting finding about these results are the cases of Pakistan and Turkey. Pakistan was included in the top 10 nations with the highest production of publications but was not included in the most cited category. Conversely, Turkey reached the tenth rank in terms of citations but, with only five documents, fell short of being included among the most productive countries regarding publications. After analyzing the number of documents and citations, which, according to Yang et al. [56], seems to be an efficient way to evaluate the impact of a country regarding a specific research area, a bibliographic coupling of the countries was carried out. In general terms, this type of analysis establishes a relationship between papers when they have indicators in common [57].

**Figure 10:**
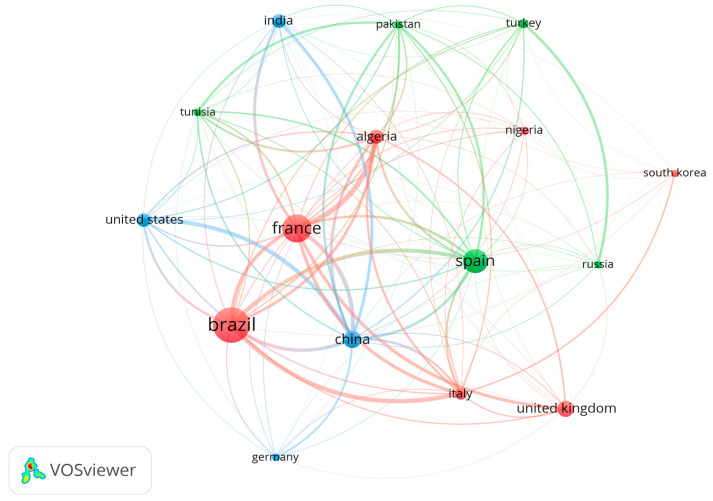
Network of countries with at least 5 documents and 100 citations.

For this, we considered a minimum of five documents per country and at least 100 citations, which yielded 16 countries and resulted in the network displayed in Figure 10. The connections between countries are based on citations, whereas the size of the circles represents the extent of the contribution of each nation. All of them are distributed in three different clusters distinguished by color: the first cluster is in red (seven countries), the second cluster in green (five countries), and the third cluster in blue (four countries). It can be noticed in the figure that Brazil leads the ranking in terms of citations, which indicates this country’s impact on this field of study. Finally, as was intended by other authors using a similar approach [8,11], this assessment aims to facilitate scientific collaboration and the sharing of work and ideas among researchers.

### 3.4. Most Relevant Authors

The analysis detected 840 different authors for the 240 publications considered in this assessment. A similar method to that used in the analysis of outcomes by country was conducted to analyze the authors: the first parameter to determine the influence of authors in this research field was the number of documents published, which yielded 760 authors with only one publication each, which represents 90% of the total number of researchers. Hence, to ensure a more precise selection, researchers with four or more articles were included, as indicated in Table 4. The primary contributor produced seven publications, followed by another scholar with six. Subsequently, four other authors produced five papers each, and finally two researchers with four publications each were identified (Table 4).

Considering that the number of citations received by authors is another way to measure their impact on a specific scientific topic, the second parameter assessed to determine the most relevant researchers were citations. The top nine authors in terms of citations are included in Table 4, which takes into account those with more than 200 citations. Additionally, the average citations are included in the list as part of the analysis without influencing the positions of the rankings.

In the table, it can be observed that four authors (Savastano Jr. H., Toledo Filho R. D., Ardanuy M., and Claramunt J.) are present in both of the rankings considered for evaluation. In terms of publications, the list is led by Savastano Jr. H., who is just one document ahead of the second position. In contrast, concerning citations, Toledo Filho R. D. secured the top position (1052 citations), surpassing the second most cited author (788 citations) by 25%. Wrapping up these results, an interesting finding is Ghavami K., who achieved the highest average citation score, and finally England G.L. and Scrivener K., the only authors in the citation ranking with a single publication each, indicating a noteworthy contribution to this field of study on their part.

**Figure 11:**
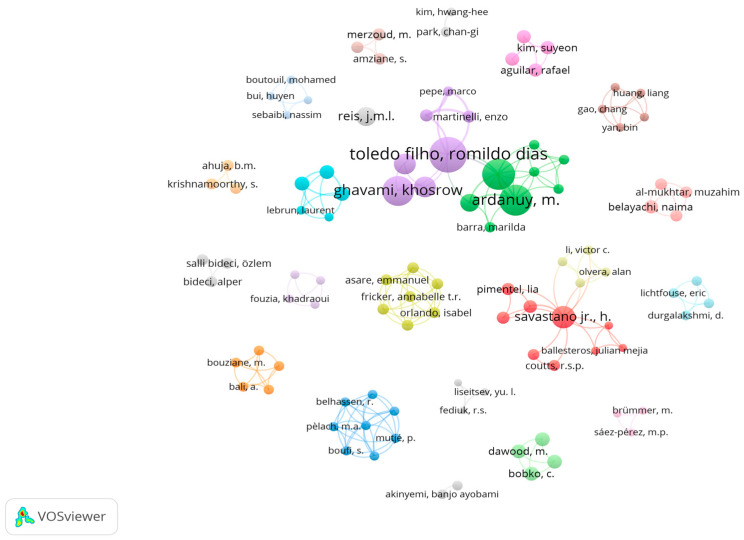
Co-authorship by authors with one document and at least 50 citations.

Having analyzed both publication counts and citations individually, a network of co-authorship by author was created in order to study the alliances between the different authors involved in the assessment. For this, a minimum number of one document with at least 50 citations per author was considered. The resulting network visualization is represented in Figure 11. Through this analysis, a total of 102 authors meeting the aforementioned criteria were recognized. This co-authorship network exposed distinct small groups of interconnected links, where the connections are established on the basis of citations.

**Figure 12:**
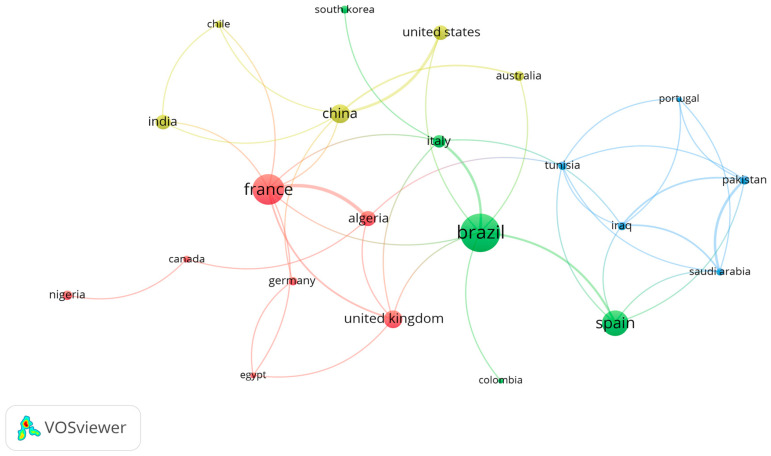
Co-authorship by countries with two documents and at least 50 citations.

The clusters revealed that, notwithstanding the distinct groupings, the network hints at a certain synergy of collaborative work between scholars. Therefore, to discern those collaborative efforts in terms of countries, an additional network considering a criterion of two documents and 50 citations is shown in Figure 12. In this case, each node represents a country, and the cooperation between them is indicated by the connection lines linking each node to the next. Furthermore, the size of the nodes corresponds to the number of citations, whereas the line thickness indicates the total link strength [58].

From this mapping, a total of 22 countries were identified, organized into four different scientific communities, as follows: Cluster 1 (red), Cluster 2 (green), Cluster 3 (blue), and Cluster 4 (yellow). The red cluster has the highest number of countries, with seven (Algeria, Canada, Egypt, France, Germany, Nigeria, and United Kingdom), while the others contain five countries each. Hence, concerning co-authorship, despite Brazil having more citations, France exhibits a stronger connection with the highest link strength, as depicted in Figure 12.

### 3.5. Most Relevant Documents

Mapping the documents according to their citation number is an additional strategy that could lead to the determination of the impact of each paper on a particular scientific topic [37,56]. Thus, two different parameters were considered to evaluate the articles from the dataset: local citations (LCs) and global citations (GCs). On one side, GCs refer to the total citations a paper obtains from every publication included in a determined source, such as Scopus in this instance. Conversely, LCs describe how many citations a document acquires from other papers within the specified research field [59]. Hence, a top 10 ranking of documents was established. Firstly, Table 5 includes a compilation based on global citations, and subsequently, a similar approach was applied for local citations, resulting in Table 6.

By making this distinction, it was possible not only to discern the influence of a specified document and its impact across other disciplines but also to determine which of those papers provide a substantial core of knowledge for the subject under investigation [60]. A significant finding derived from both tables is that the first four publications in the list of LCs also appear in the GC ranking, demonstrating their considerable contribution to this research field. An examination of the titles of these publications exposes a consistent emphasis on studying the behavior, mechanical properties, and durability implications associated with the of incorporation vegetable fibers as a form of reinforcement in composite materials. This observation establishes a crucial direction, namely, identifying keywords, which will be further analyzed in the following section.

### 3.6. Keyword Co-Occurrence

Keywords are crucial components within the field of literature research, providing insights into a specific scientific topic [61]. Nevertheless, given the potential diversity in terminology used to define the same concept, it becomes essential to standardize the words used for more accurate and consistent results [62]. For this purpose, (i) *singular forms were replaced by their corresponding plural form* (e.g., *mechanical property by mechanical properties*); and (ii) *fiber, fibre, fibers, and fibres were standardized as fibers*. Hence, after the standardization, the co-occurrence of author keywords was carried out, representing the keywords that commonly are written next to the abstract [63]. Table 7 compiles the top ten keywords identified in the analyzed dataset.

**Figure 13:**
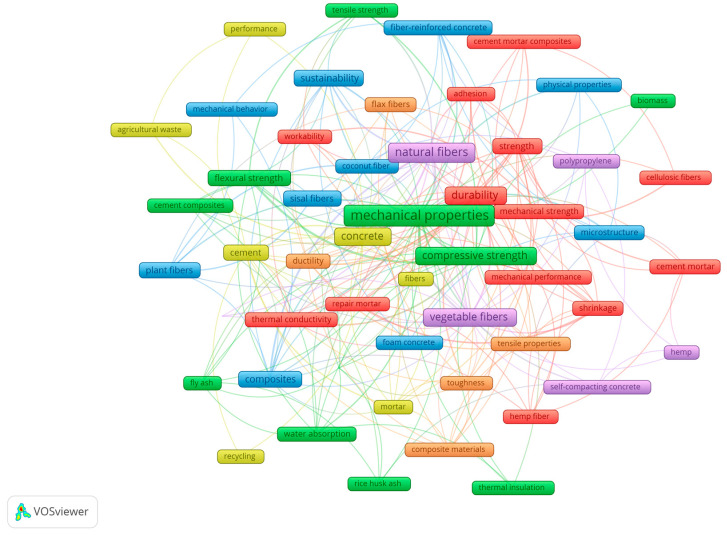
Co-occurrence of author keywords by network visualization, considering at least 3 occurrences.

In addition, considering a full counting method (wherein the weight of each co-occurrence link is considered equal) and stipulating a minimum of three occurrences per keyword, a co-occurrence network was developed, resulting in 52 keywords. Initially, Figure 13 displays the network visualization classified in six different clusters denoted by colors and distributed as follows: Cluster 1 (red) with 13 keywords, Cluster 2 (green) with 11 keywords, Cluster 3 (blue) with 10 keywords, Cluster 4 (yellow) with 7 keywords, Cluster 5 (purple) with 6 keywords, and finally Cluster 6 (orange) with 5 keywords. In this case, the size of the frame represents the occurrence of each concept, suggesting that the bigger the box, the higher the impact of the keyword on this research field.

**Figure 14:**
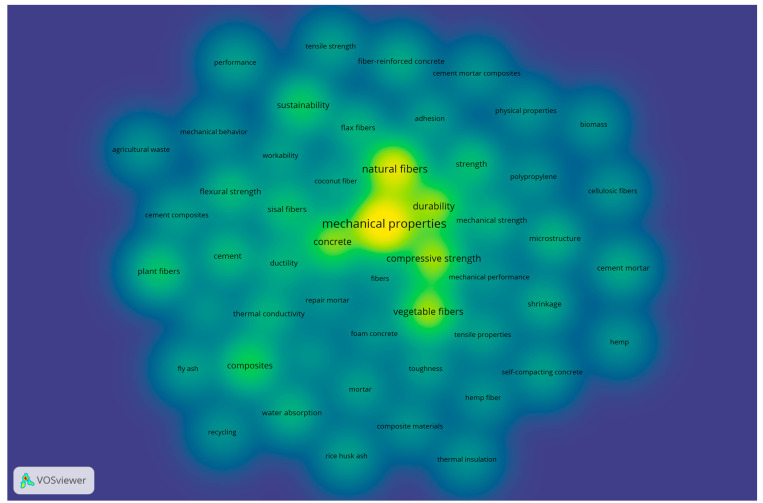
Co-occurrence of author keywords by density visualization, considering at least 3 occurrences.

Alternatively, Figure 14 represents a density visualization similar to the network previously described in Figure 13; however, in this map, the color of the label represents the density of each keyword in a color range from blue to green to yellow, where keywords closer to yellow indicate a higher number of occurrences, which is another method to graphically interpret a bibliometric map [36]. In both the network and density visualizations, a notable emphasis on the keyword “mechanical properties” is evident. This observation aligns with the findings presented in Table 7, indicating a research focus pursued by the writers in this field of study. The results obtained not only demonstrate the concerns of the scholars within the topic but should also help new authors select the appropriate keywords to identify former publications in the subject area [56].

## 4. Findings and Discussion

The principal sources of publications, as well as the most productive countries, influential authors, and significant documents, were determined by a scientometric evaluation carried out on published works on vegetable fibers in cement composites. Consequently, the main documents acquired in terms of global citations and local citations, including other studies intrinsically cited in these, were considered in a brief literature review of this scientific topic.

From a general perspective, the papers considered in this review portray vegetable fibers as potential reinforcements for inclusion in cementitious matrices due to their environmental viability and mechanical characteristics, suggesting that VFs represent a sustainable alternative in the construction field; however, authors also acknowledge certain drawbacks in this area of study: the challenge of achieving a uniform distribution of the fibers in the mix and, more importantly, ensuring their long-term durability in the cementitious matrix.

On the other hand, the scientometric analysis revealed a need for new publication trends, as it highlighted a persistent consistency in the existing topics over time. This finding presents an area of opportunity for further exploration in this review. Therefore, the proposed review is structured into four subsections: Types of Vegetable Fibers, Alternatives to Improve Durability, Mechanical Behavior, and Advanced Applications of Vegetable Fibers in Construction.

### 4.1. Types of Vegetable Fibers

**Figure 15:**
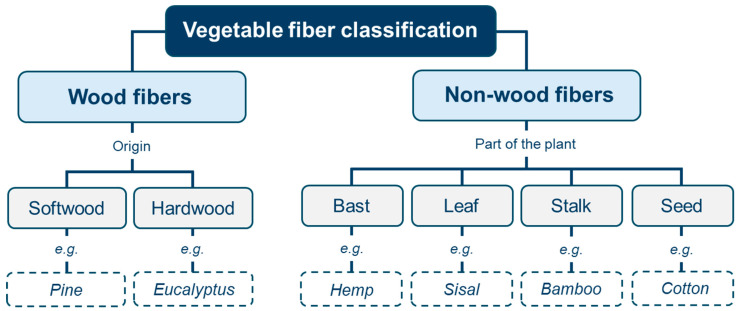
Vegetable fiber classification considered in this study, proposed by Ardanuy et al. [64].

Several types of vegetable fibers have been identified as favorable reinforcements for cement-based materials. These fibers can be divided into different groups, with categorization varying among researchers; yet, for the purpose of this study, the classification proposed by Ardanuy et al. [64] will be considered. This classification organizes VFs in two main groups: wood fibers and non-wood fibers. Wood fibers, categorized according to their origin, include softwood and hardwood fibers. On the other hand, non-wood fibers are further subdivided into four subgroups based on the part of the plant from whence they are extracted, namely into bast fibers, leaf fibers, stalk fibers, and seed fibers [64,65,66]. Figure 15 visually represents the classification described.

In the branch of wood classification, there has been extensive investigation regarding softwood fibers, particularly focusing on pine fibers obtained from the paper industry as kraft pulp. These fibers have been incorporated as reinforcement in cement mortar [67] and directly in cement paste. For the latter, one study utilized a dosage of a 10/1 cement/fiber mass ratio [68], while another utilized a 4% fiber volume fraction in the paste [69]. Similarly, hardwood fibers were examined by Savastano Jr. et al. [70], who analyzed eucalyptus pulp waste and its influence as a reinforcement for cement composites designed for roofing applications.

Regarding non-wood categorization, various plants have been contemplated as a source of bast fibers. Within this class, previous authors examined different types of fibers, such as kenaf [71] or jute [72], as a reinforcement for concrete, as well as hemp [73] for cement mortars. Likewise, flax fibers were utilized in cement composites [74] and even within geopolymer mixtures [75].

Among leaf fibers (LFs), one of the most explored plants in this scientific field is sisal, as previously noted in the Keyword Co-occurrence section, where this type of fiber was identified as a recurring topic. For instance, sisal fibers have been utilized in composites based on ordinary Portland cement (OPC) with a range of fiber mass percentages from 4 to 12% [76], or, in terms of volume fraction, with 2% [70] and 3% [77] of fibers. Additionally, these fibers have been included as a reinforcement in geopolymer matrices, with a fiber content of 1% by weight [78]. In the same vein, among LFs, numerous types of plants have been deemed suitable sources of fibers in this scientific topic; we can highlight banana as a source of kraft-pulped fibers [79] or curauá fibers [80], both in cementitious composites, as well as pineapple [81], raffia [78], and corn husk [82] fibers in geopolymer matrices. Furthermore, among LFs, a final plant of note is date palm, studied in metakaolin-based mortars [73] and as a reinforcement for concrete [83].

Turning the focus to stalk fibers (SFs), bamboo has frequently been considered by researchers, with bamboo fibers utilized in diverse applications, either incorporated into geopolymer composites [84], within a mixture of cement mortars [68], or integrated as a component in concrete [72]. Additionally, studies have explored bamboo as a replacement for steel reinforcement in concrete elements, thereby demonstrating the potential of this plant [85]. Other types of SFs explored in the literature are sugar cane bagasse [86,87] in cementitious composites and wheat straw, either reinforcing cement composites [86] or as an alternative in lightweight aggregate concrete with building isolation purposes [88], not to mention diss fibers, which have been used as a reinforcement for “green concrete” [89] or in the binder of cement and metakaolin mortars; this final application has been mirrored by including alfa fibers in mortar formulation [73]. Finally, among SFs, sweet sorghum fibers should be mentioned; these have been included in geopolymer pastes [90].

The final group among the non-wood category are seed fibers, of which there are two main sources: coconut and cotton. In the case of coconut fibers, a common term recognized in the literature is coir, representing the fibers obtained from coconut husk [78]. Different applications of coir have been identified, including reinforcement for concrete [72,87], as a component of OPC-based mortars [77], within cement composites intended for roofing purposes [70], or to reinforce a geopolymer matrix [78]. Correspondingly, studies have similarly incorporated cotton fibers in mixtures for geopolymers, seeking to enhance the mechanical and thermal properties of these type of composites [91,92], as well as adding cotton fibers to cement paste to reinforce cement mortars [67]. Moreover, a less conventional case included in this category is luffa, despite its fibers not being obtained from the seed but from the dried fruit. Alshaaer et al. [93] studied these peculiar fibers in a mixture intended for cement geopolymers based on metakaolin.

Overall, both wood and non-wood fibers showed favorable outcomes as reinforcements in cement-based materials, demonstrating their potential application in the building sector. Nevertheless, their use is conditioned by several factors, such as the availability of these materials according to the region, economic considerations, the inherent properties of the fibers, and, clearly, the intended purpose of the material. Finally, it is crucial to emphasize the importance of the dosage, type, and length of fibers in the matrix, as they are the determining factors for fiber-reinforced composites to achieve optimal properties, as highlighted by several authors [64,71,86].

### 4.2. Alternatives to Improve Durability

While incorporating VFs might enhance both the mechanical behavior and durability of composite materials, the degree of improvement is highly dependent on different factors, such as the type of fiber, treatments, content, or even the mixture. Nevertheless, as has been mentioned in this paper, the main drawback regarding vegetable fibers remains their durability within cementitious matrices, particularly due to the alkaline nature of the matrix, which deteriorates the VFs and further complicates the cement paste–fiber bond [94]. Two approaches for improving the durability of VF in such composites have been studied. The first aims to modify the inherent composition of the matrix, reducing alkali components, while the second focuses directly on the fibers, employing chemical and physical treatments to increase their stability in the mix and thereby enhancing the durability of the composite [18,64,67].

Regarding the first approach, several studies explored the possible modification of the matrix, with the incorporation of pozzolanic additions arising as a common strategy [64]. Following this line of work and working with kraft pulp fiber-reinforced composites, Mohr et al. [95] investigated the effectiveness of substituting a portion of the cement mass with different additions, including silica fume, blast furnace slag, fly ash, metakaolin, and blends of raw and calcined diatomaceous earth and volcanic ash. The outcomes demonstrated that the durability of the composite was most likely enhanced due to the reduction in calcium hydroxide content and the stabilization of alkali content.

Similarly, a partial replacement of cement with fiber-reinforced mortars was analyzed by Toledo Filho et al. [77], who substituted 10% and 40% of OPC by weight with silica fume and blast furnace slag, respectively. The results showed that composites’ embrittlement and strength loss were mitigated by the presence of silica fume; however, blast furnace slag did not exhibit the same effect, failing to decrease the embrittlement of the mortars. Nonetheless, within the same study, another method was evaluated to modify the matrix, involving an accelerated carbonation of cement composites reinforced with VFs. The study found that an environment rich in CO_2_ is a promising alternative to improve the durability of the composite with time.

Regarding the second approach, a wide range of VF treatments have been considered in the literature, with some of these procedures outlined here. Claramunt et al. [67] applied a hornification technique, achieving an irreversible effect on fibers through four drying and rewetting cycles (using an oven at 60 °C). This treatment did not alter the strength of the VFs and was able to reduce water retention and increase dimensional stability [64,67]. Similarly, Bouasker et al. [88] dried straw particles at 60 °C for 72 h in the oven and applied them as a lightweight aggregate. In line with treatments related to temperature modification, Soltan et al. [80] treated curauá fibers in 80 °C water for 18 h and then dried them at 90 °C for an additional 12 h, whereas Sellami et al. [89] boiled diss fibers for 4 h and washed them to remove organic substances, similarly to the method followed by Sawsen et al. [74], who boiled flax fibers for 5 min and then rinsed and dried them at an ambient temperature for 2 days, seeking to remove the released extractives prior to the fibers being included in cement composites.

In addition, other authors have explored alternative techniques related to the application of alkali treatments directly to the fibers. Seeking to enhance fiber–matrix adhesion, Belkadi et al. [73] immersed vegetable fibers (date palm, diss, alfa, and hemp) in a calcium hydroxide solution with a concentration of 0.73% for one hour at 20 °C, followed by drying them in an oven at 45 °C. Likewise, Ramaswamy et al. [72] previously treated VFs in a sodium hydroxide solution (pH 11) for 28 days as a reinforcement for concrete. They found good enough results in terms of the stability of the fibers in concrete.

Moreover, Kriker et al. [83] analyzed the durability of date palm fiber-reinforced concrete and the influence of three different alkaline solutions on this material: calcium hydroxide, sodium hydroxide and Lawrence solution. The results obtained demonstrated that the calcium hydroxide attack was diffuse and relatively uniform in comparison with that of sodium hydroxide, which presented a localized mechanism of attack, whereas the fibers in Lawrence solution suffered a degradation process similar to what occurs in real-environment cementitious materials, suggesting how VFs could deteriorate when incorporated into cement-based matrices. A different procedure was conducted by Sawsen et al. [74], who subjected flax fibers to a water-repellent chemical substance for 2 h, followed by drying them at an ambient temperature for an additional 2 h, seeking to decrease VF water absorption and improve the consistency of the mixture.

Furthermore, besides the previously illustrated alternatives, scholars have undertaken other procedures. These include manually debundling the fibers [80] or separating them by mechanical means [71] in order to achieve a uniform distribution within the mixture and thereby a more consistent composite material. To conclude this section, it is important to note that only for the purposes of this review, alternative methods to improve durability were given a somewhat different category. Nonetheless, it is frequently found in the literature that researchers apply multiple methods and even combine them to accomplish better results.

### 4.3. Mechanical Behavior

In the present study, diverse intrinsic technical properties of vegetable fibers, particularly their notable tensile strength, have been addressed as significant advantages for their inclusion as reinforcements in cementitious materials. Indeed, the keyword co-occurrence analysis outlined “mechanical properties” as the topic with the highest occurrence in this field of study. For this reason, this section evaluates some results obtained by researchers regarding the mechanical behavior exhibited by cement composites when VFs are applied and also considers specific outcomes related to the performance of these materials.

It is now well established by a variety of research papers that fiber incorporation produces mechanical and physical improvements in composites [96,97,98]. In particular, studies have demonstrated that the addition of fibers enhances the flexural and tensile strength of cement-based materials [99,100]. For instance, J.M.L. Reis [87] attained an increase in the flexural properties of coconut fiber-reinforced concrete in comparison with unreinforced concrete. Remarkably, this improvement was even slightly higher than that achieved with an analogous concrete reinforced with glass and carbon fiber. A better performance was also achieved regarding fracture toughness and fracture energy when comparing coconut and sugar cane bagasse fiber-reinforced concrete with conventional concrete.

These outcomes can be associated with the flexural results observed by Elsaid et al. [71] for kenaf fiber-reinforced concrete. The material exhibited a ductile failure mode characterized by a well-distributed pattern of cracks and greater energy absorption when compared to conventional concrete. A noteworthy assertion was made by Sellami et al. [89], who showed that within fiber composites, during compression and flexural tests, VFs endure tensile stress more effectively when it originates parallel to the fibers.

Related to these studies, Ramaswamy et al. [72] explored jute, coir, and bamboo fibers as concrete reinforcements, accomplishing an increase of over 25% in terms of impact strength, and thereby increased ductility, versus plain concrete. Likewise, Silva et al. [101] found that, in general terms, geopolymer matrices reinforced by different VFs, including sweet sorghum, wool, cotton, sisal, and coir, increased not only in flexural (454%) but also tensile (111%) and compressive strength (53%) compared to non-reinforced matrices, clearly turning the composite into a more ductile material.

Referring to alternative features, another interesting outcome obtained by the research group of Ramaswamy [72] was a decrease in the shrinkage characteristics (around 50–70% compared to conventional concrete) of VF-reinforced concrete. Conversely, Soltan et al. [80] evaluated other physical properties of curauá fiber cementitious composites, finding that, in terms of thermal behavior, this type of composites exhibited lower thermal conductivity and diffusivity than a fiber-less cement/fly ash mortar and even compared to a cement composite reinforced with synthetic fibers (polyvinyl alcohol). The novel composite achieved a higher specific heat capacity, which makes this material an effective option for building purposes.

**Table 5:** Ranking of documents in terms of global citations (GCs).

Ranking	Title	Authors	Year	Journal	GC	DOI	
1	Cellulosic fiber reinforced cement-based composites: A review of recent research	Mònica Ardanuy, Josep Claramunt, Romildo Dias Toledo Filho	2015	*Construction and Building Materials*	441	10.1016/j.conbuildmat.2015.01.035	[64]
2	Bamboo as reinforcement in structural concrete elements	Khosrow Ghavami	2005	*Cement and Concrete Composites*	390	10.1016/j.cemconcomp.2004.06.002	[85]
3	Development of vegetable fibre–mortar composites of improved durability	Romildo D Tolêdo Filho, Khosrow Ghavami, George L England, Karen Scrivener	2003	*Cement and Concrete Composites*	360	10.1016/S0958-9465(02)00018-5	[77]
4	Fracture and flexural characterization of natural fiber-reinforced polymer concrete	J.M.L. Reis	2006	*Construction and Building Materials*	245	10.1016/j.conbuildmat.2005.02.008	[87]
5	The hornification of vegetable fibers to improve the durability of cement mortar composites	Josep Claramunt, Mònica Ardanuy, José Antonio García-Hortal, Romildo Dias Tolêdo Filho	2011	*Cement and Concrete Composites*	173	10.1016/j.cemconcomp.2011.03.003	[67]
6	Mechanical properties of kenaf fiber reinforced concrete	A. Elsaid, M. Dawood, R. Seracino, C. Bobko	2011	*Construction and Building Materials*	162	10.1016/j.conbuildmat.2010.11.052	[71]
7	Natural fibers as reinforcement additives for geopolymers—A review of potential eco-friendly applications to the construction industry	Guido Silva, Suyeon Kim, Rafael Aguilar, Javier Nakamatsu	2020	*Sustainable Materials and Technologies*	152	10.1016/j.susmat.2019.e00132	[101]
8	Plant fibre reinforced cement components for roofing	Holmer Savastano Jr., Vahan Agopyan, Adriana M. Nolasco, Lia Pimentel	1999	*Construction and Building Materials*	126	10.1016/S0950-0618(99)00046-X	[70]
9	Improvement of mechanical properties of green concrete by treatment of the vegetals fibers	A. Sellami, M. Merzoud, S. Amziane	2013	*Construction and Building Materials*	116	10.1016/j.conbuildmat.2013.05.073	[89]
10	Physical Characterization of Natural Straw Fibers as Aggregates for Construction Materials Applications	Marwen Bouasker, Naima Belayachi, Dashnor Hoxha, Muzahim Al-Mukhtar	2014	*Materials*	113	10.3390/ma7043034	[88]

**Table 6:** Ranking of documents in terms of local citations (LCs).

Ranking	Title	Authors	Year	Journal	LC	DOI	
1	Cellulosic fiber reinforced cement-based composites: A review of recent research	Mònica Ardanuy, Josep Claramunt, Romildo Dias Toledo Filho	2015	*Construction and Building Materials*	21	10.1016/j.conbuildmat.2015.01.035	[64]
2	Improvement of mechanical properties of green concrete by treatment of the vegetals fibers	A. Sellami, M. Merzoud, S. Amziane	2013	*Construction and Building Materials*	13	10.1016/j.conbuildmat.2013.05.073	[89]
3	The hornification of vegetable fibers to improve the durability of cement mortar composites	Josep Claramunt, Mònica Ardanuy, José Antonio García-Hortal, Romildo Dias Tolêdo Filho	2011	*Cement and Concrete Composites*	12	10.1016/j.cemconcomp.2011.03.003	[67]
4	Mechanical properties of kenaf fiber reinforced concrete	A. Elsaid, M. Dawood, R. Seracino, C. Bobko	2011	*Construction and Building Materials*	8	10.1016/j.conbuildmat.2010.11.052	[71]
5	Fiber-matrix interactions in cement mortar composites reinforced with cellulosic fibers	Mònica Ardanuy, Josep Claramunt, José Antonio García-Hortal, Marilda Barra	2011	*Cellulose*	8	10.1007/s10570-011-9493-3	[68]
6	Introducing a curauá fiber reinforced cement-based composite with strain-hardening behavior	Daniel G. Soltan, Patricia das Neves, Alan Olvera, Holmer Savastano Junior, Victor C. Li	2017	*Industrial Crops and Products*	7	10.1016/j.indcrop.2017.03.016	[80]
7	Effect of vegetable and synthetic fibers on mechanical performance and durability of Metakaolin-based mortars	Ahmed Abderraouf Belkadi, Salima Aggoun, Chahinez Amouri, Abdelhamid Geuttala, Hacene Houari	2018	*Journal of Adhesion Science and Technology*	7	10.1080/01694243.2018.1442647	[73]
8	Durability of date palm fibres and their use as reinforcement in hot dry climates	A. Kriker, A. Bali, G. Debicki, M. Bouziane, M. Chabannet	2008	*Cement and Concrete Composites*	6	10.1016/j.cemconcomp.2007.11.006	[83]
9	Behaviour of concrete reinforced with jute, coir and bamboo fibres	H.S. Ramaswamy, B.M. Ahuja, S. Krishnamoorthy	1983	*International Journal of Cement Composites and Lightweight Concrete*	5	10.1016/0262-5075(83)90044-1	[72]
10	Effect of flax fibers treatments on the rheological and the mechanical behavior of a cement composite	Chafei Sawsen, Khadraoui Fouzia, Boutouil Mohamed, Gomina Moussa	2015	*Construction and Building Materials*	5	10.1016/j.conbuildmat.2014.12.091	[74]

**Table 7:** Top ten author keywords based on co-occurrence.

**Ranking**	**Keyword**	**Occurrence**
1	Mechanical properties	41
2	Natural fibers	28
3	Compressive strength	19
4	Vegetable fibers	19
5	Durability	18
6	Concrete	17
7	Composites	12
8	Sustainability	10
9	Plant fibers	9
10	Sisal fibers	8

### 4.4. Advanced Applications of Vegetable Fibers in Construction

As discussed in this paper, the research field on vegetable fibers is wide, complex, multidisciplinary, and continuously evolving. Hence, this section elucidates other potential applications of VFs within the construction sector and discusses emerging types of fibers that have garnered recent attention among scholars.

Based on their unique hygrothermal properties, vegetable fibers can be used to create products with densities similar to materials such as concrete, wood, or plastic [102], making them a viable alternative for insulation purposes. For instance, Kinnane et al. [103] studied hemp–lime concrete walls and their acoustic absorption capabilities, demonstrating that they significantly enhance sound insulation properties. Similarly, the research group of El-yahyaoui et al. [104] analyzed the effect of doum fibers on unfired clay-based bricks, proving that the specimens exhibited an improvement in insulation characteristics, thereby enhancing their thermal behavior for building purposes. Additionally, Liuzzi et al. [105] evaluated the hygric and thermal properties of clay-based plasters incorporating olive fibers obtained from olive tree pruning. Their findings indicated that fiber incorporation reduced the density and increased the porosity of the plasters, thereby enhancing insulation by lowering the thermal conductivity. Furthermore, their study revealed an increase in moisture adsorption for plasters with a higher fiber content.

Moreover, regarding “non-conventional” vegetable fibers, researchers have begun to explore alternative plant sources in recent studies, thereby expanding the scope within the scientific field. In this line, açai fibers arise as a solid candidate. The authors implied that their interweave tendency may exhibit good fiber–matrix adhesion [106]. De Azevedo et al. [107] demonstrated that using açai fibers with Portland cement mortars is feasible, specifically on wall covering plasters and even for reinforcing small structural points. On the other hand, the research work conducted by de Oliveira et al. [108] focused on castor oil-based polyurethane composites reinforced with açaí waste. The reinforcement improved both the thermal and impact resistance of the composites, turning this material into an attractive option for building insulation.

In addition, Juncus acutus fibers were studied by Omrani et al. [109], particularly for their incorporation in clay–sand composites, revealing a feasible utilization of this material for lightweight construction purposes. Acceptable results were obtained in terms of this material’s mechanical and thermal properties; therefore, it is an appropriate composite for improving energy efficiency in the building sector. An unconventional source of plant-based fibers found in the literature is the Himalayan Nettle (*Girardinia diversifolia* L.), specifically in a study proposed by Maitra et al. [110], who developed nonwoven sound absorption panels from nettle fibers and demonstrated their efficiency for acoustic purposes.

These supplementary studies offer further evidence of the diverse applications and benefits of vegetable fibers in enhancing the performance of construction materials, highlighting the significant potential for continued innovation and development in this dynamic research area. The extensive panorama of vegetable fibers, which may be obtained from different sources, such as diverse worldwide plants, agricultural production, and even waste, delineates a broad and prolific field that continually motivates researchers to explore new avenues and advancements.

## 5. Future Outlooks

After conducting this study, it was possible to identify gaps, research opportunities, and future recommendations for this scientific field. Some of these will be presented in general terms below.

### 5.1. Life Cycle Assessment

To provide a more comprehensive understanding of the environmental benefits of vegetable fibers in the building sector, future studies should include Life Cycle Assessments (LCAs) of VF cementitious composites. Recent publications have begun to incorporate this type of analysis in their research [11], highlighting the need for a standardized and precise methodology for conducting LCAs in this field, among other requirements. An original case regarding this approach was conducted by Alcivar-Bastidas et al. [111], who analyzed how the reutilization of hydroxide sodium solution affects the alkalinization of abaca fibers as incorporation in cement mortars. The results demonstrated an outstanding reduction in carbon footprint and an improvement in tensile strength. Thus, conducting further investigations with this methodology presents a significant opportunity for future research in the scientific field of vegetable fibers.

### 5.2. Industry Integration

The gap between research into and the industrial integration of VFs within building materials is an established reality. Transitioning these resources into a scalable production process requires significant development before they can be adopted into conventional construction materials. This development should focus on cost-effective methods for the large-scale manufacturing of VF cement composites, ensuring, for instance, quality control, standardization, and specifications for their application in construction projects. Therefore, encouraging a collaborative framework involving researchers, stakeholders, and policymakers is crucial to promote and facilitate their widespread adoption and utilization.

### 5.3. Further Durability Enhancement

It has been clearly demonstrated in this study that enhancing durability is a primary focus among authors in this scientific field. However, despite the extensive research already conducted, this remains a persistent trend that future studies should further explore. In this line, research is currently analyzing accelerated aging tests, advanced coating, and novel additives (to mention a few) aiming to improve the long-term durability of VFs in cement composites for sustainable construction. For instance, an approach that has not been mentioned in this analysis is the incorporation on nanomaterials, which have demonstrated an ability to enhance the durability and interfacial properties of concrete, leading to improved resistance against environmental degradation [112]. Nevertheless, further advancements are needed to establish standardized methodologies and scalable solutions, ensuring the practical application of these innovations in real-world construction.

### 5.4. CO_2_ Storage of VF Cement Composites

Previous research has established that a significant amount of CO_2_ can be stored by cement-based materials, for instance, through accelerated carbonation curing, which according to Ashraf [113] exhibits both the highest and fastest CO_2_ storage in concrete. Although this process requires that specimens be precast, given that the cement matrix hardens rapidly, it suggests that precast cement-bonded cellulose fiber boards [114] could be a viable option for carbon capture.

A particular case was studied by Rakhsh Mahpour et al. [115], who utilized nonwoven flax fabrics as a reinforcement in lime-based composites exposed to high CO_2_ conditions, noting improved mechanical properties and revealing that the fibers enhanced matrix carbonation. Using a different approach, Gunn et al. [116] investigated carbon sequestration as part of the curing procedure for mortars incorporating rice husk biochar, finding an enhancement in both strength and CO_2_ sequestration.

These findings reveal a promising scenario for VFs and highlight the need for additional research to further advance this approach and promote more sustainable practices through construction materials.

### 5.5. Machine Learning Approaches

Machine learning (ML) techniques have become widespread across various disciplines, including engineering and construction. For instance, they have been used to predict the compressive strength of fiber-reinforced concrete by using learning algorithms [117]. A notable case is the work conducted by Kueh et al. [118], which integrated experimental work with statistical and ML-based approaches to forecast the sound and mechanical properties of concrete and composites using pineapple leaf fibers. These models demonstrated promising results, ensuring practical reliability and reducing the need for extensive laboratory work.

While there have already been preceding papers analyzing different models to make these predictions [119], many have focused on industrial fibers, such as steel fiber-reinforced cementitious composites [120,121] or basalt fiber-reinforced concrete [122], to mention some further cases. However, this methodology is still a novel approach for optimizing the properties of VF cement composites. Notably, the research group of Natesan et al. [123] explored the possibilities of incorporating ML with natural fiber-reinforced composites to detect and predict damage in real-time conditions. These outcomes underscore the potential role of ML approaches in identifying complex patterns and relationships that are not easily noticeable through traditional experimental procedures, paving the way for more efficient and innovative applications of VF composites.

## 6. Conclusions

Utilizing the Scopus database, the scientific subject concerning vegetable fibers in cement composites was explored. We obtained a wide dataset published from 1983 to 2024. The open-source tools Bibliometrix R-package and VOSviewer were selected for the scientometric analysis. The results revealed a progressive increase in the number of documents over the last decade. Despite the analysis being conducted in the early part of the year, the data collected thus far indicate an analogous trend for 2024. On the other hand, based on the evaluation of the most relevant sources, a low concentration of documents per source was shown: 90 of the 121 journals included reported only one publication. The most influential journal was *Construction and Building Materials*, covering 39% of the publications considered in the scientometric analysis.

From the perspective of scientific production by country, China leads the ranking in terms of the number of documents, while Brazil has the first position in terms of the number of citations. Conversely, among the most relevant authors, Savastano Jr. H. presented the highest number of publications, whereas Toledo Filho R.D. was identified as the most-cited author within the analyzed data. The distinction between global citations (GCs) and local citations (LCs) was applied for the most relevant documents, allowing us to recognize that the research group of Ardanuy and their paper “Cellulosic fiber reinforced cement-based composites: A review of recent research” was the most influential document, leading both the GC and LC rankings.

Keyword co-occurrence analysis revealed a significant emphasis on mechanical properties, which is the most studied topic by scholars in the field. Additionally, the section allowed us to point out some other interests pursued by authors, establishing a research focus that involves certain technical properties, fiber categories, and special attention paid to the durability of these composites, as well as regular references to sustainability. Considering the most relevant documents identified in this assessment and some of the keywords retrieved, a literature review was undertaken and separated into four subsections: (i) Types of Vegetable Fibers, (ii) Alternatives to Improve Durability, (iii) Mechanical Behavior, and (iv) Advanced Applications of Vegetable Fibers in Construction.

Therefore, in general terms, the findings obtained in this bibliometric analysis underscore the complexity of this scientific field, revealing multidisciplinary research alliances, a general overview of the field, and the research avenues pursued by the scholars. The current study led to the following conclusions:

(1) The primary classification of VFs includes wood fibers and non-wood fibers. However, these main branches can be broken down into a wide variety of VF categories according to the origin of the fibers and the part of the plant they are derived from. This classification provides clear comprehension for future research and practical applications in cement composites.
(2) Alternative methods to improve durability were divided into two general approaches: the modification of the cement matrix and treatments applied directly to the VFs. Several alternatives were identified, highlighting the incorporation of additions and cement replacements to alkalize the matrix, while among the treatments, different physical and chemical methods were found, such as hornification, boiling, and adding alkaline solutions (calcium or sodium hydroxide) to the fibers. Future research might focus on refining these treatments and exploring their long-term effects while developing new alternatives for VFs in composites, ensuring their practical viability in construction.
(3) The application of VFs results in an improvement in the mechanical behavior of cement-based materials, enhancing properties such as flexural, tensile, and compressive strength and ensuring a more ductile performance of the composites, a research line that remains widely studied in the field.
(4) The advanced applications of vegetable fibers in construction further highlighted the wider potential of VFs in building materials, particularly in insulation and acoustic applications. This underscored the use of “non-conventional” fibers such as acai, Juncus acutus, and Himalayan Nettle as potential alternatives. The results suggest that further applications are still open and pending study, offering opportunities for the exploration of new, sustainable solutions in the construction industry.
(5) Future outlooks were identified for this research field, suggesting avenues such as Life Cycle Assessment (LCA), industry integration, further durability enhancement, CO_2_ absorption, and machine learning approaches to VFs in cementitious composites. These directions offer guidelines for future studies focusing on assessing the environmental impact of composites, improving VF usage in the construction industry, and encouraging machine learning to optimize and predict the service performance of materials. Promoting collaborative research between researchers and the industry will be essential to make these innovations become practical and accessible.

## Figures and Tables

**Table 1:** Filters applied for data retrieval from Scopus.

Filter	Limited to
Timespan	1983 to 2024
Subject area	Engineering Materials Science Environmental Science Energy Agricultural and Biological Sciences Earth and Planetary Sciences Chemistry Chemical Engineering
Document type	Article Review
Publication stage	Final
Source type	Journal
Language	English

**Table 2:** Journals with the most publications in the field, along with their CiteScore and Impact Factor values until 2023.

Journal	Documents	CiteScore	Impact Factor
*Construction and Building Materials*	34	13.8	7.4
*Cement and Concrete Composites*	13	18.7	10.8
*Materials*	12	5.8	3.1
*Case Studies in Construction Materials*	7	7.6	6.5
*Industrial Crops and Products*	7	9.5	5.6
*Journal of Building Engineering*	7	10.0	6.7
*Buildings*	6	3.4	3.1
*Sustainability* (Switzerland)	6	6.8	3.3
*Journal of Cleaner Production*	5	20.4	9.8
*Fibers*	4	7.0	4.0

**Table 3:** Ranking of countries in terms of the number of publications and citations.

Ranking	Country	Documents	Citations	Average Citations
Number of documents				
1	China	38	557	14.66
2	France	30	1468	48.93
3	Brazil	29	2339	80.66
4	Algeria	23	371	16.13
5	India	23	349	15.17
6	Italy	20	243	12.15
7	Spain	13	1049	80.69
8	United States	11	347	31.55
9	Pakistan	10	126	12.60
10	United Kingdom	6	496	82.67
Number of citations				
1	Brazil	29	2339	80.66
2	France	30	1468	48.93
3	Spain	13	1049	80.69
4	China	38	557	14.66
5	United Kingdom	6	496	82.67
6	Algeria	23	371	16.13
7	India	23	349	15.17
8	United States	11	347	31.55
9	Italy	20	243	12.15
10	Turkey	5	170	34.00

**Table 4:** Ranking of authors in terms of the number of publications and citations.

Ranking	Author	Country	Documents	Citations	Average Citations
Number of documents					
1	Savastano Jr. H.	Brazil	7	425	60.71
2	Toledo Filho R.D.	Brazil	6	1052	175.33
3	Ardanuy M.	Spain	5	788	157.60
4	Claramunt J.	Spain	5	788	157.60
5	Ferrara G.	Italy	5	125	25.00
6	Martinelli E.	Italy	5	125	25.00
7	Mydin M.A.O.	Malaysia	4	24	6.00
8	Pepe M.	Italy	4	78	19.50
Number of citations					
1	Toledo Filho R.D.	Brazil	6	1052	175.33
2	Ardanuy M.	Spain	5	788	157.60
3	Claramunt J.	Spain	5	788	157.60
4	Ghavami K.	Brazil	2	750	375.00
5	Savastano Jr. H.	Brazil	7	425	60.71
6	England G.L.	United Kingdom	1	360	360.00
7	Scrivener K.	Switzerland	1	360	360.00
8	Reis J.M.L.	Brazil	3	307	102.33
9	García-Horta J.A.	Spain	2	266	133.00

## Data Availability

The raw data supporting the conclusions of this article will be made available from the corresponding author on request.

## References

[1] Klemm D., Heublein B., Fink H., Bohn A. (2005). Cellulose: Fascinating Biopolymer and Sustainable Raw Material. Angew. Chem. Int. Ed..

[2] Ding G.K.C. (2008). Sustainable Construction—The Role of Environmental Assessment Tools. J. Environ. Manag..

[3] Fiore V., Scalici T., Di Bella G., Valenza A. (2015). A Review on Basalt Fibre and Its Composites. Compos. Part B Eng..

[4] Meyer C. (2009). The Greening of the Concrete Industry. Cem. Concr. Compos..

[5] Luca A., Antonio G., Scalia Giada L., Fata Concetta Manuela L., Rosa M. (2023). Life Cycle Assessment of a New Industrial Process for Sustainable Construction Materials. Ecol. Indic..

[6] McLellan B.C., Williams R.P., Lay J., Van Riessen A., Corder G.D. (2011). Costs and Carbon Emissions for Geopolymer Pastes in Comparison to Ordinary Portland Cement. J. Clean. Prod..

[7] Laverde V., Marin A., Benjumea J.M., Rincón Ortiz M. (2022). Use of Vegetable Fibers as Reinforcements in Cement-Matrix Composite Materials: A Review. Constr. Build. Mater..

[8] Mohamed S., Elemam H., Seleem M.H., Sallam H.E.-D.M. (2024). Effect of Fiber Addition on Strength and Toughness of Rubberized Concretes. Sci. Rep..

[9] Chen H., Chow C.L., Lau D. (2022). Developing Green and Sustainable Concrete in Integrating with Different Urban Wastes. J. Clean. Prod..

[10] Takagi H., Asano A. (2008). Effects of Processing Conditions on Flexural Properties of Cellulose Nanofiber Reinforced “Green” Composites. Compos. Part A Appl. Sci. Manuf..

[11] Manso-Morato J., Hurtado-Alonso N., Revilla-Cuesta V., Skaf M., Ortega-López V. (2024). Fiber-Reinforced Concrete and Its Life Cycle Assessment: A Systematic Review. J. Build. Eng..

[12] Brandt A.M. (2008). Fibre Reinforced Cement-Based (FRC) Composites after over 40 Years of Development in Building and Civil Engineering. Compos. Struct..

[13] Bendahane K., Belkheir M., Mokaddem A., Doumi B., Boutaous A. (2023). Date and Doum Palm Natural Fibers as Renewable Resource for Improving Interface Damage of Cement Composites Materials. Beni-Suef Univ. J. Basic Appl. Sci..

[14] Singh J.K., Rout A.K., Kumari K. (2021). A Review on Borassus Flabellifer Lignocellulose Fiber Reinforced Polymer Composites. Carbohydr. Polym..

[15] Murillo M., Sánchez A., Gil A., Araya-Letelier G., Burbano-Garcia C., Silva Y.F. (2024). Use of Animal Fiber-Reinforcement in Construction Materials: A Review. Case Stud. Constr. Mater..

[16] Jiang D., An P., Cui S., Xu F., Tuo T., Zhang J., Jiang H. (2018). Effect of Leaf Fiber Modification Methods on Mechanical and Heat-Insulating Properties of Leaf Fiber Cement-Based Composite Materials. J. Build. Eng..

[17] Cai M., Takagi H., Nakagaito A.N., Katoh M., Ueki T., Waterhouse G.I.N., Li Y. (2015). Influence of Alkali Treatment on Internal Microstructure and Tensile Properties of Abaca Fibers. Ind. Crops Prod..

[18] Alcivar-Bastidas S., Petroche D.M., Martinez-Echevarria M.J. (2023). The Effect of Different Treatments on Abaca Fibers Used in Cementitious Composites. J. Nat. Fibers.

[19] Donnini J., Corinaldesi V., Nanni A. (2016). Mechanical Properties of FRCM Using Carbon Fabrics with Different Coating Treatments. Compos. Part B Eng..

[20] Asim M., Uddin G.M., Jamshaid H., Raza A., Rehman Tahir Z.U., Hussain U., Satti A.N., Hayat N., Arafat S.M. (2020). Comparative Experimental Investigation of Natural Fibers Reinforced Light Weight Concrete as Thermally Efficient Building Materials. J. Build. Eng..

[21] Hakamy A., Shaikh F.U.A., Low I.M. (2015). Thermal and Mechanical Properties of NaOH Treated Hemp Fabric and Calcined Nanoclay-Reinforced Cement Nanocomposites. Mater. Des..

[22] Kabir M.M., Wang H., Lau K.T., Cardona F. (2012). Chemical Treatments on Plant-Based Natural Fibre Reinforced Polymer Composites: An Overview. Compos. Part B Eng..

[23] Achour A., Ghomari F., Belayachi N. (2017). Properties of Cementitious Mortars Reinforced with Natural Fibers. J. Adhes. Sci. Technol..

[24] Xie H., Zhang Y., Wu Z., Lv T. (2020). A Bibliometric Analysis on Land Degradation: Current Status, Development, and Future Directions. Land.

[25] Noyons E.C.M., Moed H.F., Luwel M. (1999). Combining Mapping and Citation Analysis for Evaluative Bibliometric Purposes: A Bibliometric Study. J. Am. Soc. Inf. Sci..

[26] Khan K., Ahmad W., Amin M.N., Nazar S. (2022). Nano-Silica-Modified Concrete: A Bibliographic Analysis and Comprehensive Review of Material Properties. Nanomaterials.

[27] Donthu N., Kumar S., Mukherjee D., Pandey N., Lim W.M. (2021). How to Conduct a Bibliometric Analysis: An Overview and Guidelines. J. Bus. Res..

[28] Wallin J.A. (2005). Bibliometric Methods: Pitfalls and Possibilities. Basic. Clin. Pharma Tox.

[29] He C., Zhang S., Liang Y., Ahmad W., Althoey F., Alyami S.H., Javed M.F., Deifalla A.F. (2022). A Scientometric Review on Mapping Research Knowledge for 3D Printing Concrete. Materials.

[30] Chadegani A.A., Salehi H., Yunus M.M., Farhadi H., Fooladi M., Farhadi M., Ebrahim N.A. (2013). A Comparison between Two Main Academic Literature Collections: Web of Science and Scopus Databases. Asian Soc. Sci..

[31] Visser M., Van Eck N.J., Waltman L. (2021). Large-Scale Comparison of Bibliographic Data Sources: Scopus, Web of Science, Dimensions, Crossref, and Microsoft Academic. Quant. Sci. Stud..

[32] Afgan S., Bing C. (2021). Scientometric Review of International Research Trends on Thermal Energy Storage Cement Based Composites via Integration of Phase Change Materials from 1993 to 2020. Constr. Build. Mater..

[33] Baas J., Schotten M., Plume A., Côté G., Karimi R. (2020). Scopus as a Curated, High-Quality Bibliometric Data Source for Academic Research in Quantitative Science Studies. Quant. Sci. Stud..

[34] Lilargem Rocha D., Tambara Júnior L., Marvila M., Pereira E., Souza D., De Azevedo A. (2022). A Review of the Use of Natural Fibers in Cement Composites: Concepts, Applications and Brazilian History. Polymers.

[35] Aria M., Cuccurullo C. (2017). Bibliometrix: An R-Tool for Comprehensive Science Mapping Analysis. J. Informetr..

[36] Van Eck N.J., Waltman L. (2010). Software Survey: VOSviewer, a Computer Program for Bibliometric Mapping. Scientometrics.

[37] De La Torre Bayo J.J., Martín-Lara M.Á., Calero Hoces M., Sánchez Castillo P.M., Pula H.J., Zamorano M. (2023). Management of Used COVID-19 Personal Protective Equipment: A Bibliometric Analysis and Literature Review. Appl. Sci..

[38] Ejidike C.C., Mewomo M.C. (2023). Benefits of Adopting Smart Building Technologies in Building Construction of Developing Countries: Review of Literature. SN Appl. Sci..

[39] Nalbandian K.M., Carpio M., González Á. (2021). Analysis of the Scientific Evolution of Self-Healing Asphalt Pavements: Toward Sustainable Road Materials. J. Clean. Prod..

[40] Cândido L.F., Lazaro J.C., Freitas E Silva A.O.D., Barros Neto J.D.P. (2023). Sustainability Transitions in the Construction Sector: A Bibliometric Review. Sustainability.

[41] Ferreira G.M.G., Cecchin D., Azevedo A.R.G., Valadão I.C.R.P., Costa K.A., Silva T.R., Ferreira F., Amaral P.I.S., Huther C.M., Sousa F.A. (2021). Bibliometric Analysis on the Use of Natural Fibers in Construction Materials. Agron. Res..

[42] Riccaboni M., Verginer L. (2022). The Impact of the COVID-19 Pandemic on Scientific Research in the Life Sciences. PLoS ONE.

[43] Nicola M., Alsafi Z., Sohrabi C., Kerwan A., Al-Jabir A., Iosifidis C., Agha M., Agha R. (2020). The Socio-Economic Implications of the Coronavirus Pandemic (COVID-19): A Review. Int. J. Surg..

[44] Rodrigues M., Franco M., Silva R. (2020). COVID-19 and Disruption in Management and Education Academics: Bibliometric Mapping and Analysis. Sustainability.

[45] Gao J., Yin Y., Myers K.R., Lakhani K.R., Wang D. (2021). Potentially Long-Lasting Effects of the Pandemic on Scientists. Nat. Commun..

[46] Okagbue H.I., Teixeira Da Silva J.A. (2020). Correlation between the CiteScore and Journal Impact Factor of Top-Ranked Library and Information Science Journals. Scientometrics.

[47] Fernandez-Llimos F. (2018). Differences and Similarities between Journal Impact Factor and CiteScore. Pharm Pr..

[48] Quan W., Mongeon P., Sainte-Marie M., Zhao R., Larivière V. (2019). On the Development of China’s Leadership in International Collaborations. Scientometrics.

[49] Shu F., Julien C., Larivière V. (2019). Does the Web of Science Accurately Represent Chinese Scientific Performance?. J. Assoc. Inf. Sci. Technol..

[50] Zhu J., Liu W. (2020). Comparing like with like: China Ranks First in SCI-Indexed Research Articles since 2018. Scientometrics.

[51] Li N., Han R., Lu X. (2018). Bibliometric Analysis of Research Trends on Solid Waste Reuse and Recycling during 1992–2016. Resour. Conserv. Recycl..

[52] Mohammed L., Ansari M.N.M., Pua G., Jawaid M., Islam M.S. (2015). A Review on Natural Fiber Reinforced Polymer Composite and Its Applications. Int. J. Polym. Sci..

[53] García G., Cabrera R., Rolón J., Pichardo R., Thomas C. (2024). Natural Fibers as Reinforcement of Mortar and Concrete: A Systematic Review from Central and South American Regions. J. Build. Eng..

[54] Confraria H., Godinho M.M. (2015). The Impact of African Science: A Bibliometric Analysis. Scientometrics.

[55] Sooryamoorthy R. (2018). The Production of Science in Africa: An Analysis of Publications in the Science Disciplines, 2000–2015. Scientometrics.

[56] Yang H., Liu L., Yang W., Liu H., Ahmad W., Ahmad A., Aslam F., Joyklad P. (2022). A Comprehensive Overview of Geopolymer Composites: A Bibliometric Analysis and Literature Review. Case Stud. Constr. Mater..

[57] Weinberg B.H. (1974). Bibliographic Coupling: A Review. Inf. Storage Retr..

[58] Zhang X., Estoque R.C., Xie H., Murayama Y., Ranagalage M. (2019). Bibliometric Analysis of Highly Cited Articles on Ecosystem Services. PLoS ONE.

[59] Batista-Canino R.M., Santana-Hernández L., Medina-Brito P. (2023). A Scientometric Analysis on Entrepreneurial Intention Literature: Delving Deeper into Local Citation. Heliyon.

[60] Beliaeva T., Ferasso M., Kraus S., Mahto R.V. (2022). Marketing and Family Firms: Theoretical Roots, Research Trajectories, and Themes. J. Bus. Res..

[61] Li H., An H., Wang Y., Huang J., Gao X. (2016). Evolutionary Features of Academic Articles Co-Keyword Network and Keywords Co-Occurrence Network: Based on Two-Mode Affiliation Network. Phys. A Stat. Mech. Its Appl..

[62] Su H.-N., Lee P.-C. (2010). Mapping Knowledge Structure by Keyword Co-Occurrence: A First Look at Journal Papers in Technology Foresight. Scientometrics.

[63] Cancino C., Merigó J.M., Coronado F., Dessouky Y., Dessouky M. (2017). Forty Years of Computers & Industrial Engineering: A Bibliometric Analysis. Comput. Ind. Eng..

[64] Ardanuy M., Claramunt J., Toledo Filho R.D. (2015). Cellulosic Fiber Reinforced Cement-Based Composites: A Review of Recent Research. Constr. Build. Mater..

[65] Faruk O., Bledzki A.K., Fink H.-P., Sain M. (2012). Biocomposites Reinforced with Natural Fibers: 2000–2010. Prog. Polym. Sci..

[66] Jawaid M., Abdul Khalil H.P.S. (2011). Cellulosic/Synthetic Fibre Reinforced Polymer Hybrid Composites: A Review. Carbohydr. Polym..

[67] Claramunt J., Ardanuy M., García-Hortal J.A., Filho R.D.T. (2011). The Hornification of Vegetable Fibers to Improve the Durability of Cement Mortar Composites. Cem. Concr. Compos..

[68] Ardanuy M., Claramunt J., García-Hortal J.A., Barra M. (2011). Fiber-Matrix Interactions in Cement Mortar Composites Reinforced with Cellulosic Fibers. Cellulose.

[69] Mohr B.J., Nanko H., Kurtis K.E. (2005). Durability of Kraft Pulp Fiber–Cement Composites to Wet/Dry Cycling. Cem. Concr. Compos..

[70] Savastano H., Agopyan V., Nolasco A.M., Pimentel L. (1999). Plant Fibre Reinforced Cement Components for Roofing. Constr. Build. Mater..

[71] Elsaid A., Dawood M., Seracino R., Bobko C. (2011). Mechanical Properties of Kenaf Fiber Reinforced Concrete. Constr. Build. Mater..

[72] Ramaswamy H.S., Ahuja B.M., Krishnamoorthy S. (1983). Behaviour of Concrete Reinforced with Jute, Coir and Bamboo Fibres. Int. J. Cem. Compos. Lightweight Concr..

[73] Belkadi A.A., Aggoun S., Amouri C., Geuttala A., Houari H. (2018). Effect of Vegetable and Synthetic Fibers on Mechanical Performance and Durability of Metakaolin-Based Mortars. J. Adhes. Sci. Technol..

[74] Sawsen C., Fouzia K., Mohamed B., Moussa G. (2015). Effect of Flax Fibers Treatments on the Rheological and the Mechanical Behavior of a Cement Composite. Constr. Build. Mater..

[75] Silva G., Kim S., Castañeda A., Donayre R., Nakamatsu J., Aguilar R., Korniejenko K., Łach M., Mikuła J. (2018). A Comparative Study of Linen (Flax) Fibers as Reinforcement of Fly Ash and Clay Brick Powder Based Geopolymers. IOP Conference Series: Materials Science and Engineering.

[76] Savastano H., Warden P.G., Coutts R.S.P. (2003). Mechanically Pulped Sisal as Reinforcement in Cementitious Matrices. Cem. Concr. Compos..

[77] Tolêdo Filho R.D., Ghavami K., England G.L., Scrivener K. (2003). Development of Vegetable Fibre–Mortar Composites of Improved Durability. Cem. Concr. Compos..

[78] Korniejenko K., Frączek E., Pytlak E., Adamski M. (2016). Mechanical Properties of Geopolymer Composites Reinforced with Natural Fibers. Procedia Eng..

[79] Savastano H., Warden P.G., Coutts R.S.P. (2003). Potential of Alternative Fibre Cements as Building Materials for Developing Areas. Cem. Concr. Compos..

[80] Soltan D.G., das Neves P., Olvera A., Savastano Junior H., Li V.C. (2017). Introducing a Curauá Fiber Reinforced Cement-Based Composite with Strain-Hardening Behavior. Ind. Crops Prod..

[81] Correia E.A.S., Torres S.M., De Oliveira Alexandre M.E., Gomes K.C., P. Barbosa N., De Barros S.R. (2013). Mechanical Performance of Natural Fibers Reinforced Geopolymer Composites. Materials Science Forum.

[82] Musil S.S., Keane P.F., Kriven W.M., Kriven W.M., Wang J., Zhou Y., Gyekenyesi A.L., Kirihara S., Widjaja S. (2013). Green Composite: Sodium-Based Geopolymer Reinforced with Chemically Extracted Corn Husk Fibers. Ceramic Engineering and Science Proceedings.

[83] Kriker A., Bali A., Debicki G., Bouziane M., Chabannet M. (2008). Durability of Date Palm Fibres and Their Use as Reinforcement in Hot Dry Climates. Cem. Concr. Compos..

[84] Sá Ribeiro R.A., Sá Ribeiro M.G., Sankar K., Kriven W.M. (2016). Geopolymer-Bamboo Composite—A Novel Sustainable Construction Material. Constr. Build. Mater..

[85] Ghavami K. (2005). Bamboo as Reinforcement in Structural Concrete Elements. Cem. Concr. Compos..

[86] Khorami M., Ganjian E. (2011). Comparing Flexural Behaviour of Fibre–Cement Composites Reinforced Bagasse: Wheat and Eucalyptus. Constr. Build. Mater..

[87] Reis J.M.L. (2006). Fracture and Flexural Characterization of Natural Fiber-Reinforced Polymer Concrete. Constr. Build. Mater..

[88] Bouasker M., Belayachi N., Hoxha D., Al-Mukhtar M. (2014). Physical Characterization of Natural Straw Fibers as Aggregates for Construction Materials Applications. Materials.

[89] Sellami A., Merzoud M., Amziane S. (2013). Improvement of Mechanical Properties of Green Concrete by Treatment of the Vegetals Fibers. Constr. Build. Mater..

[90] Chen R., Ahmari S., Zhang L. (2014). Utilization of Sweet Sorghum Fiber to Reinforce Fly Ash-Based Geopolymer. J. Mater. Sci..

[91] Alomayri T., Shaikh F.U.A., Low I.M. (2013). Characterisation of Cotton Fibre-Reinforced Geopolymer Composites. Compos. Part B Eng..

[92] Alomayri T., Shaikh F.U.A., Low I.M. (2013). Thermal and Mechanical Properties of Cotton Fabric-Reinforced Geopolymer Composites. J. Mater. Sci..

[93] Alshaaer M., Mallouh S.A., Al-Kafawein J., Al-Faiyz Y., Fahmy T., Kallel A., Rocha F. (2017). Fabrication, Microstructural and Mechanical Characterization of Luffa Cylindrical Fibre—Reinforced Geopolymer Composite. Appl. Clay Sci..

[94] Agopyan V., Savastano H., John V.M., Cincotto M.A. (2005). Developments on Vegetable Fibre–Cement Based Materials in São Paulo, Brazil: An Overview. Cem. Concr. Compos..

[95] Mohr B.J., Biernacki J.J., Kurtis K.E. (2007). Supplementary Cementitious Materials for Mitigating Degradation of Kraft Pulp Fiber-Cement Composites. Cem. Concr. Res..

[96] Aamr-Daya E., Langlet T., Benazzouk A., Quéneudec M. (2008). Feasibility Study of Lightweight Cement Composite Containing Flax By-Product Particles: Physico-Mechanical Properties. Cem. Concr. Compos..

[97] Wei J., Gencturk B. (2018). Degradation of Natural Fiber in Cement Composites Containing Diatomaceous Earth. J. Mater. Civ. Eng..

[98] Hasan K.M.F., Horváth P.G., Alpár T. (2021). Development of Lignocellulosic Fiber Reinforced Cement Composite Panels Using Semi-Dry Technology. Cellulose.

[99] Mansur M.A., Aziz M.A. (1982). A Study of Jute Fibre Reinforced Cement Composites. Int. J. Cem. Compos. Lightweight Concr..

[100] Awwad E., Mabsout M., Hamad B., Farran M.T., Khatib H. (2012). Studies on Fiber-Reinforced Concrete Using Industrial Hemp Fibers. Constr. Build. Mater..

[101] Silva G., Kim S., Aguilar R., Nakamatsu J. (2020). Natural Fibers as Reinforcement Additives for Geopolymers—A Review of Potential Eco-Friendly Applications to the Construction Industry. Sustain. Mater. Technol..

[102] Crini G., Lichtfouse E., Chanet G., Morin-Crini N. (2020). Applications of Hemp in Textiles, Paper Industry, Insulation and Building Materials, Horticulture, Animal Nutrition, Food and Beverages, Nutraceuticals, Cosmetics and Hygiene, Medicine, Agrochemistry, Energy Production and Environment: A Review. Environ. Chem. Lett..

[103] Kinnane O., Reilly A., Grimes J., Pavia S., Walker R. (2016). Acoustic Absorption of Hemp-Lime Construction. Constr. Build. Mater..

[104] El-yahyaoui A., Manssouri I., Lehleh Y., Sahbi H., Limami H. (2024). Enhancing the Mechanical and Thermal Insulation Properties of Clay-Based Construction Materials with Neutral Carbon Footprint through the Use of Doum Fibers. Mater. Chem. Phys..

[105] Liuzzi S., Rubino C., Stefanizzi P., Petrella A., Boghetich A., Casavola C., Pappalettera G. (2018). Hygrothermal Properties of Clayey Plasters with Olive Fibers. Constr. Build. Mater..

[106] Silva T.R., De Matos P.R., Tambara Júnior L.U.D., Marvila M.T., Monteiro S.N., Azevedo A.R.G., Zhang M., Peng Z., Li B., Monteiro S.N., Soman R., Hwang J.-Y., Kalay Y.E., Escobedo-Diaz J.P., Carpenter J.S., Brown A.D. (2023). Characterization of Açaí Fibers (Euterpe Oleracea Mart.) for Application in Cement Composites. TMS Annual Meeting & Exhibition.

[107] De Azevedo A.R.G., Marvila M.T., Tayeh B.A., Cecchin D., Pereira A.C., Monteiro S.N. (2021). Technological Performance of Açaí Natural Fibre Reinforced Cement-Based Mortars. J. Build. Eng..

[108] De Oliveira B.P., Balieiro L.C.S., Maia L.S., Zanini N.C., Teixeira E.J.O., Da Conceição M.O.T., Medeiros S.F., Mulinari D.R. (2022). Eco-Friendly Polyurethane Foams Based on Castor Polyol Reinforced with Açaí Residues for Building Insulation. J. Mater. Cycles Waste Manag..

[109] Omrani H., Hassini L., Benazzouk A., Beji H., ELCafsi A. (2020). Elaboration and Characterization of Clay-Sand Composite Based on Juncus Acutus Fibers. Constr. Build. Mater..

[110] Maitra S., Sahni S., Gupta D. (2024). Nonwoven Acoustic Panels from Himalayan Nettle (*Girardinia diversifolia* L.) Fibre. Ind. Crops Prod..

[111] Alcivar-Bastidas S., Petroche D.M., Ramirez A.D., Martinez-Echevarria M.J. (2024). Characterization and Life Cycle Assessment of Alkali Treated Abaca Fibers: The Effect of Reusing Sodium Hydroxide. Constr. Build. Mater..

[112] Adesina A. (2020). Nanomaterials in Cementitious Composites: Review of Durability Performance. J. Build. Rehabil..

[113] Ashraf W. (2016). Carbonation of Cement-Based Materials: Challenges and Opportunities. Constr. Build. Mater..

[114] Monkman S., Shao Y. (2010). Integration of Carbon Sequestration into Curing Process of Precast Concrete. Can. J. Civ. Eng..

[115] Rakhsh Mahpour A., Ventura H., Ardanuy M., Rosell J.R., Claramunt J. (2023). The Effect of Fibres and Carbonation Conditions on the Mechanical Properties and Microstructure of Lime/Flax Composites. Cem. Concr. Compos..

[116] Gunn P.F.E., Onn C.C., Mo K.H., Lee H.V. (2024). Enhancing Carbon Sequestration in Cement Mortar Using High Volume Local Rice Husk Biochar Coupled with Carbonation Curing. Case Stud. Constr. Mater..

[117] Chen H., Yang J., Chen X. (2021). A Convolution-Based Deep Learning Approach for Estimating Compressive Strength of Fiber Reinforced Concrete at Elevated Temperatures. Constr. Build. Mater..

[118] Kueh A., Razali A., Lee Y., Hamdan S., Yakub I., Suhaili N. (2023). Acoustical and Mechanical Characteristics of Mortars with Pineapple Leaf Fiber and Silica Aerogel Infills—Measurement and Modeling. Mater. Today Commun..

[119] Omran B.A., Chen Q., Jin R. (2016). Comparison of Data Mining Techniques for Predicting Compressive Strength of Environmentally Friendly Concrete. J. Comput. Civ. Eng..

[120] Guo P., Meng W., Xu M., Li V.C., Bao Y. (2021). Predicting Mechanical Properties of High-Performance Fiber-Reinforced Cementitious Composites by Integrating Micromechanics and Machine Learning. Materials.

[121] Kang M.-C., Yoo D.-Y., Gupta R. (2021). Machine Learning-Based Prediction for Compressive and Flexural Strengths of Steel Fiber-Reinforced Concrete. Constr. Build. Mater..

[122] Cakiroglu C., Aydın Y., Bekdaş G., Geem Z.W. (2023). Interpretable Predictive Modelling of Basalt Fiber Reinforced Concrete Splitting Tensile Strength Using Ensemble Machine Learning Methods and SHAP Approach. Materials.

[123] Natesan R., Krishnasamy P. (2024). Fiber and Matrix-Level Damage Detection and Assessments for Natural Fiber Composites. J. Mater. Sci..

